# Protein Kinase CK1α Sustains B-Cell Receptor Signaling in Mantle Cell Lymphoma

**DOI:** 10.3389/fonc.2021.733848

**Published:** 2021-10-14

**Authors:** Sabrina Manni, Anna Fregnani, Laura Quotti Tubi, Zaira Spinello, Marco Carraro, Greta Scapinello, Andrea Visentin, Gregorio Barilà, Marco Pizzi, Angelo Paolo Dei Tos, Fabrizio Vianello, Renato Zambello, Carmela Gurrieri, Gianpietro Semenzato, Livio Trentin, Francesco Piazza

**Affiliations:** ^1^ Department of Medicine-DIMED, Hematology and Clinical Immunology Section, University of Padova, Padova, Italy; ^2^ Laboratory of Myeloma and Lymphoma Pathobiology, Veneto Institute of Molecular Medicine, Padova, Italy; ^3^ Department of Medicine-DIMED, Surgical Pathology and Cytopathology Unit, University of Padova, Padova, Italy

**Keywords:** mantle cell lymphoma, CK1α, BCR inhibitors, ibrutinib, duvelisib, targeted therapy

## Abstract

Mantle Cell Lymphoma (MCL) is still an incurable B-cell malignancy characterized by poor prognosis and frequent relapses. B Cell Receptor (BCR) signaling inhibitors, in particular of the kinases BTK and PI3Kγ/δ, have demonstrated clinically meaningful anti-proliferative effects in B cell tumors. However, refractoriness to these drugs may develop, portending a dismal prognosis. Protein kinase CK1α is an emerging pro-growth enzyme in B cell malignancies. In multiple myeloma, this kinase sustains β-catenin and AKT-dependent survival and is involved in the activation of NF-κB in B cells. In this study, we analyzed the role of CK1α on MCL cell survival and proliferation, on the regulation of BCR-related BTK, NF-κB, PI3K/AKT signaling cascades and the effects of CK1α chemical inhibition or gene silencing in association with the BTK inhibitor Ibrutinib or the PI3Kγ/δ inhibitor Duvelisib. CK1α was found highly expressed in MCL cells as compared to normal B cells. The inactivation/loss of CK1α caused MCL cell apoptosis and proliferation arrest. CK1α sustained BCR signaling, in particular the NF-κB, AKT and BTK pathways by modulating the phosphorylation of Ser 652 on CARD11, Ser 536 p65 on NF-κB, Ser 473 on AKT, Tyr 223 on BTK, as well as the protein levels. We also provided evidence that CK1α-mediated regulation of CARD11 and BTK likely implicates a physical interaction. The combination of CK1α inhibition with Ibrutinib or Duvelisib synergistically increased cytotoxicity, leading to a further decrease of the activation of BCR signaling pathways. Therefore, CK1α sustains MCL growth through the regulation of BCR-linked survival signaling cascades and protects from Ibrutinib/Duvelisib-induced apoptosis. Thus, CK1α could be considered as a rational molecular target for the treatment of MCL, in association with novel agents.

## Introduction

Mantle Cell Lymphoma (MCL) is a B-cell neoplasm - characterized by clinical and morphological variants (1) - that accounts for about 6% of all Non-Hodgkin Lymphoma (NHL) in the United States, and 7–9% in Europe (2). It is characterized by a high incidence of relapse, even after the introduction of novel drugs in the therapeutic armamentarium (3).

Recent studies have shown that MCL cells display an aberrant activation of survival signaling pathways. These include the B-Cell Receptor (BCR)-related signaling cascades, such as Phosphatidylinositide 3-Kinases/AKT (PI3K/AKT)/mammalian Target Of Rapamycin (mTOR), the Nuclear Factor kappa-light-chain-enhancer of activated B cells (NF-κB) and the Extracellular signal-Regulated Kinase (ERK) cascades, as well as signals elicited by Tumor Necrosis Factor alpha (TNFα), Hedgehog and Wingless (WNT) pathways. Moreover, the B-Cell Lymphoma 2 (BCL-2) family of apoptosis regulators have also been implicated in MCL growth (4).

In normal B cells, after the engagement of the BCR by the antigen, Immunoreceptor Tyrosine-based Activation Motifs (ITAMs) are phosphorylated and this event in turn recruits the cytosolic Src Family tyrosine Kinases (SFKs) Lyn and Spleen Tyrosine Kinase (SYK). Next, the phosphorylation of Bruton Tyrosine Kinase (BTK) on Tyr 551 leads to its autophosphorylation at Tyr 223, necessary for the full activation of this kinase. A subsequent cascade of events follows, through the activation of Phospholipase Cγ2 (PLCγ2) generation of inositol triphosphate (IP_3_) and diacylglycerol (DAG), the release of calcium and the stimulation of Protein Kinase C β (PKCβ) (5). PKCβ phosphorylates caspase recruitment domain-containing protein 11 (CARD11) on Ser 652, enabling it to recruit B cell CLL/lymphoma 10 (BCL10) and Mucosa-associated lymphoid tissue lymphoma translocation protein 1 (MALT1) into a multiprotein CARD11-BCL10-MALT1 (CBM1) complex that facilitates the activation of the NF-κB inhibitor (IκB) kinase (IKK), thereby initiating NF-κB signaling (6). Moreover, upon BCR engagement, the stimulation of the PI3K/AKT signal transduction pathway results in the activating AKT phosphorylation on Ser 473.

Ibrutinib is an oral drug, which inhibits BTK enzymatic activity and it is currently employed in the therapy of relapsed/refractory MCL. Even though Ibrutinib has shown an overall response rate of 68% (7), approximately 30% of MCL patients display a primary resistance to the drug, maybe due to a lack of normal BTK expression or presence of mutated BTK (5, 8, 9). Along with Ibrutinib, other BTK inhibitors such as Acalabrutinib and Zanubrutinib are now approved in the relapsed setting in MCL (10). Duvelisib is a PI3Kγ/δ inhibitor approved for the treatment of Chronic Lymphocytic Leukemia (LLC) and Follicular Lymphoma (FL) (11, 12). Its use in MCL is controversial and, to date, there are limited studies regarding the potential use in the therapy of MCL.

Protein kinase CK1 consists of a family of multiple isoforms with distinct biochemical characteristics. In mammalians, seven isoforms are encoded by different genes (α, β, γ1, γ2, γ3, δ and ϵ), which have highly conserved kinase domains, but differ significantly in length and primary structure of their N-terminal and C-terminal regulatory non-catalytic domains (13, 14). CK1 takes part in many cellular processes, such as stress response, DNA damage response, cell cycle progression, spindle-dynamics and chromosome segregation and apoptosis (13, 15).

The isoform α (CK1α) is encoded by the *CSNK1A1* gene, mapping on chromosome 5q32. It regulates a broad range of cellular processes, and it modulates several signaling pathways such as PI3K/AKT, NF-κB, WNT/β-catenin (13, 16). We and others, recently demonstrated that CK1α sustains multiple myeloma (MM) cell growth (17, 18) positively regulating β-catenin and AKT signaling (17) and supporting a prosurvival autophagy (19, 20). CK1α was found highly expressed in MM patients in a large microarray data set series (17). This protein level is reduced by lenalidomide in MM cells and stromal cells, indicating a role in therapy. A recent study has described CK1α as a tumor growth-propeller in Diffuse Large B-cell Lymphoma (DLBCL), by regulating NF-κB signaling intensity (21). In T cells, after antigen receptor engagement, CK1α physically interacts with CARD11 (21) and phosphorylates MALT1 on ser 562, being important for the assembly of the CBM1 complex and the fully activation of NF-κB signaling (22). Several highly potent CK1-specific, ATP competitive, small molecule inhibitors have been identified. D4476 {4-[4(2,3-Dihydro-1,4-benzodioxin-6-yl)-5-(2-pyridinyl)-1H-imidazol-2-yl] benzamide} is one of the best commercially available cell permeant inhibitor specific for CK1 isoforms α and δ (23). Very recently, the compound A-51 was discovered as a novel dual inhibitor of CK1α and the kinase CDK7/9 with an anti-leukemic effect in preclinical models (24).

Given the putative role of CK1α in signal transduction pathways crucial for MCL, in the present study, we investigated its function in MCL downstream of the BCR signaling. We aimed at i) assessing the effect of CK1α inactivation on the proliferation, survival, sensitivity to therapeutic agents and intracellular signaling of MCL cells, ii) evaluating CK1α involvement in the BCR cascade and in sustaining MCL cell “addiction” to the BCR; iii) evaluating if CK1α inhibition could empower BCR inhibitors (such as Ibrutinib and Duvelisib) cytotoxicity for the treatment of MCL.

## Material and Methods

### Patients and Cell Cultures

PBMC, MCL cell lines Jeko-1, Rec-1, Granta-519 and primary PBMC from patients were isolated and cultured as previously described (25). Malignant and healthy B cells were isolated with EasySep™ kits (STEMCELL Technologies, USA), after achieving informed consent according to the declaration of Helsinki, with approval of the internal Institutional Board (protocol # 4089/AO/17). The clinical features of the patients analyzed are described in **Table 1**.

**Table 1:** Clinical and pathological features of MCL cases analyzed.

Patient	Age/sex	Stage/MIPI	IHC					IgH/CCND1 FISH	karyotype	outcome
			CD5	CD20	CD23	Ciclina D1	Ki-67%			
MCL#1	68/M	IVA	+	+	–	+	10-15	nd	nd	A
MCL#2	66/F	IVA	+	+	–	+	nd	nd	nd	A
MCL#3	70/M	IVB	+	+	+	nd	nd	nd	normal	D
MCL#4	76/F	IVA/MIPI 5.9 (*intermediate risk*)	–	+	nd	+	nd	+	normal	A
MCL#5	78/M	IVA	+	+	–	+	40	–	normal	D
MCL#6	51/F	IVB/MIPI 6.6 (*high risk*)	–	nd	–	+	nd	–	normal	A
MCL#7	74/M	IVA/MIPI 9 (*high risk*)	+	+	–	+	40	+	complex*	A
MCL#8	81/F	IVA	+	+	–	+	nd	nd	normal	D
MCL#9	81/M	IVA	+	++	–	+	nd	–	nd	D
MCL#10	65/M	IVA/MIPI6.1 (*intermediate risk*)	+	–	–	+	10	–	normal	A
MCL#11	55/F	IVA	+	++	–	+	nd	nd	nd	A
MCL#12	74/F	IVA/MIPI 6.4 (*high risk*)	+	+	–	+	nd	+	Complex**	A
MCL#13	72/F	IVA/MIPI 6.1 (*intermediate risk*)	+	+	–	+	nd	nd	complex***	D
MCL#14	71/M	nd	+	+	–	+	nd	+	complex****	D
MCL#15	85/F	IVA	+	+	–	+	nd	–	normal	D
MCL#16	71/M	IVA/MIPI 6.1 (*intermediate risk*)	+	+	–	+	nd	+	normal	A
MCL#17	79/M	IVA	+	++	+	+	nd	+	complex°	D
MCL#18	68/M	IVB/MIPI *high risk*	+	++	–	+	nd	+	nd	A
MCL#19	58/M	IVA/MIPI 5.6 (*intermediate risk*)	+	+	–	+	nd	+	complex°°	A
MCL#20	61/M	IVA	+	+	–	+	nd	+	normal	A

MIPI, Mantle cell International Prognostic Index; IHC, immunohistochemistry; nd, not determined; A, alive; D, death. M, male; F, female.

*43,XY,add(1)(q4)?,+3,del(6)(q13),-8,-9,der(10)t(10;13)(p13;q12),del(11)(q13),-13,-13,-14,+mar[13]/46,XY[12].ish add(1)(IGH++,CCND1++),der(11)t(11;14)(CCND1+,IGH+),add(?)(IGH+,CCND1+)[10].

**47XX,+3,del(6),t(10;15),t(11;14).

***62,XX,-X,-3,-5,+8,-12,-16,-17,dup(17)(p11p13),-18,+21,i(22)(q10),+mar[cp6]/46,XX[4].

****hyperdiploid karyotype due to a low number of mytosis with del(17)(p13) by FISH.

ᵒ42,X,-Y,+3,der(6;17)(q12;p11)?t(6;19)(q12;p13),i(8)(q10),der(11)t(11;14)(q13;q32),-13,-14,-15,add(15)(p13),add(20)(q13)[22]/46,XY[6].nuc ish(CCND1,IGH)x3(CCND1conIGHx2)[233/300].

ᵒᵒ43,C,-Y,+der(3)del(3)(p22)add(3)(q26),dic(4;6)(p16;q13),add(7)(p21),i(8)(q10),-10,t(11;14)(q13;q32),-13.der(15)t(10;15)(q21;q21),-20,-r[cp14]/46,XY[14].

### Chemicals

IPTG was from Sigma-Aldrich, (Italy); Ibrutinib, Duvelisib, Bortezomib and Z-VAD-FMK was from Selleck chemicals (USA); D4476 (4-[4-(2,3-Dihydro-1,4-benzodioxin-6-yl)-5-(2-pyridinyl)-1*H*-imidazol-2-yl]benzamide) was from abcam (UK). Working dilution of D4476 was prepared using Fugene VI reagent (Promega, Italy).

### Evaluation of Growth and Apoptosis

Cell viability was measured through Trypan blue exclusion dye assay. Apoptosis was assessed by Annexin V/Propidium Iodide (PI) staining (IMMUNOSTEP, Spain) and FACS analysis as in (17).

### Cell Cycle Analysis

It was performed as described in (17).

### Assessment of Drug Concentration-Effect and Calculation of the Combination Index

Jeko-1 and Granta-519 cells were plated into 96 well plates in 100 μl media. D4476, Ibrutinib, and Duvelisib were added at different concentrations for 72 h alone or in combination. Cell viability was analyzed with 3-(4,5-dimethylthiazol-2-yl)- 2,5-diphenyltetrazolium bromide (MTT) and the CI was calculated as in (26).

### Immunofluorescence

It was performed as in (26) with the following antibodies: anti-CK1α (abcam, UK), secondary Alexa-Fluor 594-conjugated goat anti-rabbit (Life technologies, Italy), MALT1 and CARD11, (Santa Cruz Biotechnology, Inc (USA) using Alexa-Fluor 488 conjugated anti-mouse. Specimens were mounted in Vectashield medium with DAPI (Vector Laboratories, USA) and analyzed using Zeiss LSM 700 E90 confocal microscope, oil objective 63x (Italy).

### Western Blot

WB was performed as described (27). Antibodies used were the following: CK1α, PARP, Mcl1, total β-catenin, Ser 473 AKT, total AKT, Ser 176/180, Ser 177/181 IKKα/α, total IKKα, IKKβ, Ser 536 NF-κB p65, Ser 652 CARD11, Tyr 223 BTK, total BTK, Ser 32 Ikbα, BCL10 (Cell signaling Technology, MA, USA); GAPDH (Ambion, USA), β-actin (Sigma-Aldrich, Italy); p21 (Becton Dickinson, Italy); Caspase 3 (Enzo Life Science, UK); total p65 (abcam, UK), DEPTOR (Millipore, Itlay); CARD11 and BTK for immunoprecipitation (Santa Cruz Biotechnology, Inc; Italy). Images were acquired using the Image Quant LAS 500 chemiluminescence detection system (GE Healthcare, USA).

### Immunoprecipitation

Cells were lysed in immunoprecipitation (IP) buffer containing Tris 50mM, NaCl 150 mM, Triton 1%, NP-40 1%, EDTA 2mM, phosphatases and proteases inhibitors (Life technologies and SIGMA Aldrich). 500-800 µg of total protein lysates were precleared for 30 min at 4°C with anti-mouse or anti-goat agarose beads (e-bioscience, Italy), according to the antibody used for the IP. After centrifugation, the supernatant was incubated overnight 4°C with the dedicated antibody (BTK, CARD11 or CK1α) or with the corresponding pre-immune serum. Agarose beads were added to the protein suspension for 2h at 4°C. After extensive washes in IP buffer the immunocomplex was resuspended in Laemmli buffer with β-mercaptoethanol and processed for WB analysis.

### RNA Interference

RNA interference (RNAi) was performed with nucleofection of double strand (ds) siRNA and by the generation of inducible shRNA MCL cell clones. Nucleofection was performed by electroporation of Jeko-1 using the Amaxa system (Lonza, Rockland, Inc.) with 100 pmol of scrambled siRNAs or CK1α targeting siRNAs ON-TARGET plus SMARTpool siRNA (Thermo Scientific, USA), as described (17) through the nucleofector solution R with the program A-023. Cells were harvested at 48h after nucleofection. Cells were simultaneously electroporated with a fluorescent oligonucleotide (siGLO green). The transfection rate, evaluated by FACS analysis, has always been around 95%. To perform shRNA lentiviral transduction, Jeko-1 and Granta-519 cells were transduced with the IPTG inducible lentiviral particles carrying *CSNK1A1*-specific shRNA (pLKO_IPTG_3XLacO, Sigma-Aldrich, Italy) with the sequence TRCN0000006044. 2x10^4^ cells were infected with a multiplicity of infection of 12, using the spinfection method, in the presence of 8µg/ml polybrene (Sigma-Aldrich, Italy). Puromycin selection (1µg/ml) was initiated two days after transduction. A titration curve for puromycin resistance was performed for Jeko-1 and Granta-519 cells by an Antibiotic Kill Curve Assay. Once a cellular clone was established, to induce CK1α silencing, cells were incubated with 500/1000µM IPTG every two/three day for a total of one (in the case of Granta-519) or two (in the case of Jeko-1) weeks, time lapse in which the best knockdown efficacy was obtained.

### Quantitative Real-Time PCR

Performed as in (25) using the QuantStudio 5 detection system (Applied Biosystem, CA, USA) with the QuantStudio™ Design and Analysis Software v.1.4.3. The primers used are the following: *BTK* Forward 5’-3’ GGGGTTTGCTCAGACTGTCC and Reverse 5’-3’ AATCACTGCGGCCATAGCTT; *RELA* Forward 5’-3’ CCCCACGAGCTTGTAGGAAAG and Reverse 5’-3’ CCAGGTTCTGGAAACTGTGGAT; *BIRC3* Forward 5’-3’ GACAGGAGTTCATCCGTCAAG and Reverse 5’-3’ TTCCACGGCAGCATTAAT; *GAPDH* Forward 5’-3’ AATGGAAATCCCATCACCATCT and Reverse 5’-3’ CGCCCCACTTGATTTTGG.

### Statistical Analysis

Data were examined for their statistical significance with the two-tail unpaired Student’s *t* test or ANOVA analysis of variance with *post-hoc* corrections. Values were considered statistically significant at *p* values below 0.05.

## Results

### CK1α Is Overexpressed in MCL Samples Compared to Healthy B Cells

We analyzed CK1α mRNA expression in different subtypes of B cell derived cancers (through the publicly available database Oncomine), finding that *CSNK1A1* mRNA is highly expressed in MCL human samples as compared to normal B lymphocytes (www.oncomine.org, “Basso Lymphoma” data set). Thus, we evaluated CK1α protein level in purified B lymphocytes from healthy controls, MCL patients and the MCL cell lines Jeko-1, Granta-519 and Rec-1.

**Figure 1:**
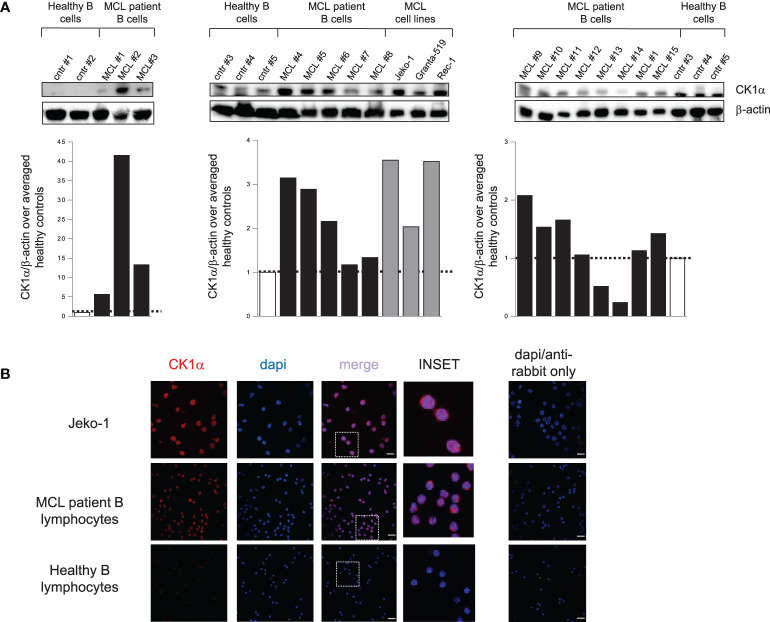
CK1α protein is overexpressed in MCL patients and cell lines compared to controls. **(A)** WB analysis (upper panel) and the corresponding densitometric values (lower panel) of CK1α protein expression in purified B lymphocytes from 5 healthy buffy coats (white bars), from 15 MCL patients (black bars) and from the MCL cell lines Jeko-1, Granta-519 and Rec-1 (grey bars). β-actin was used as a loading control. For each blot, the densitometric values of CK1α in MCL patients and cell lines are reported as arbitrary units over averaged healthy controls within the blot. **(B)** CK1α cellular distribution along with DAPI staining in Jeko-1 cell lines, a MCL patient sample and healthy lymphocytes from buffy coat. CK1α is detected by red fluorescence and nuclei by DAPI. On the right panel staining with only Alexa fluor 594-conjugated goat anti-rabbit secondary antibody merged with dapi is shown. Images were collected with 63X magnification, oil objective. The insert shows a detail of the image. Scale bars = 25µm.

Immunoblot and densitometric analysis revealed that all the MCL cell lines and most of MCL patients overexpressed CK1α when compared to normal B lymphocytes, even if with some variability between different MCL samples (**Figure 1A**). Among a total of fifteen MCL patients analyzed, eleven showed CK1α overexpression in malignant B cells at variance from what seen in B cells from healthy controls. CK1α was overexpressed in 100% of patients in **Figure 1A** left, in 80% of patients in **Figure 1A** middle, and in 50% of patients in **Figure 1A** right. On average, 77% of MCL patients and 100% of the MCL cell lines tested showed overexpressed CK1α at the protein level.

To study the localization of the kinase inside B cells, we performed a confocal immunofluorescence analysis in purified B lymphocytes from a healthy control, a MCL patient and in Jeko-1 cells. CK1α localized both in the cytoplasm and in the nucleus of Jeko-1 and MCL patient cells while it was restricted in cytoplasm in healthy B lymphocytes (**Figure 1B**). These data, which are similar to what we observed in another B cell malignancy like MM (17), suggest an abnormal localization of CK1α in MCL cells.

### CK1α Sustains MCL Growth and Proliferation

**Figure 2:**
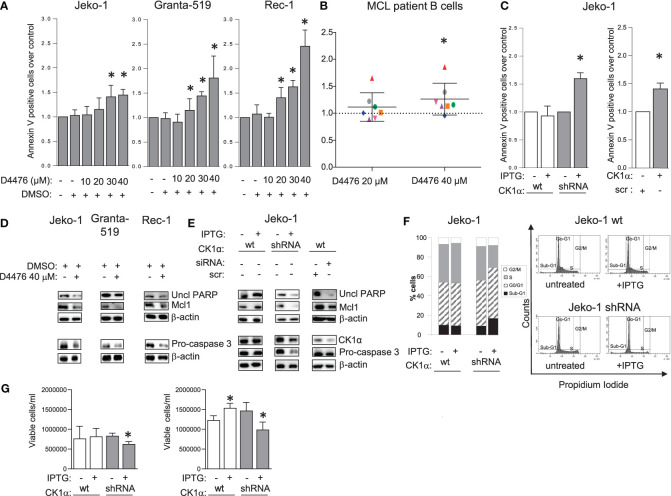
CK1α sustains MCL cell survival and proliferation. Histogram showing annexin V positive cells of MCL cell lines Jeko-1 Granta-519 and Rec-1 **(A)**, 7 independent MCL patients B cells **(B)** treated with different concentration of D4476 for 48h **(A)** or 24h **(B)**, Jeko-1 IPTG inducible *CSNK1A1* directed shRNA clone (named CK1α shRNA) and Jeko-1 wt treated with IPTG 500µM for 13 days (**C** left panel) or Jeko-1 cells electroporated with *CSNK1A1* directed siRNAs for 48h (**C** right panel). Data are expressed as mean ± SD of n=3 **(A)**, n=7 **(B)** n=4 (**C** left panel) and n=3 (**C** right panel) independent experiments. * indicates *p* < 0.05 compared to the untreated cell population. The silencing efficacy and the expression of anti-apoptotic proteins are showed in **(D, E)** In WB panels, β-actin was used as loading control. Uncl PARP= Uncleaved PARP. **(F)** cell cycle distribution with PI staining and FACS analysis of the same cells as in **(C)**. Left: bar graph representing the average of four independent experiments of the sub-G1 (black), G0/G1 (striped, gray), S (grey) and G2/M (white) phases of the cell cycle. Right: representative FACS histogram plots. **(G)** Trypan blue negative (viable) Jeko-1 wt (white bars) and Jeko 1 shRNA cellular clone (grey bars) treated with IPTG 500µM for 6 days (left panel) or 13 days (right panel). Data are expressed as mean ± SD of n=3 experiments (wt and shRNA clone, left panel), n=4 experiments (wt Jeko-1 right panel) and n=8 experiments (Jeko-1 shRNA clone, right panel). * indicates *p* < 0.05 compared to the corresponding untreated cell population.

To examine if CK1α is essential for MCL cell growth, Jeko-1, Granta-519, Rec-1 and B cells isolated from 7 MCL patients, were treated with increasing concentrations of the CK1 chemical inhibitor D4476 or with the vehicle DMSO. Apoptosis was investigated through Annexin V (AV) and PI labelling and FACS analysis. Treatment with DMSO alone did not produce any significant changes in cell viability compared to untreated cells. All the MCL cell lines were sensitive to D4476, starting from the 20 µM concentration for Granta-519 and Rec-1 cells and 30 µM for Jeko-1 (**Figures 2A**, **S1**). Even if with some variability, malignant B cells from most of MCL patients proved to be sensitive to D4476 (6 out of 7) used at 40 µM. Of note, B cells from 3 patients were responsive also to the 20 µM D4476 concentration (**Figure 2B**). We previously showed that D4476 is not toxic for healthy B lymphocytes at any concentration used (17).

To validate the results obtained with the chemical inhibitor of CK1 and to investigate the specific role of the α isoform, we used RNAi to knockdown CK1α protein in Jeko-1 cells. CK1α knockdown was obtained through the generation of IPTG-inducible *CSNK1A1* specific shRNAs or with electroporation of double-strand (ds) *CSNK1A1* directed siRNAs (**Figures 2C**, **S1**). The number of apoptotic cells was assessed by FACS analysis of AV/PI positive cells, which showed increased apoptotic rate upon silencing. To exclude off-target effects induced by IPTG *per se*, wt Jeko-1 cells were treated with the same concentration of IPTG and this treatment did not increase apoptosis. The proapoptotic effect of CK1α inhibition or silencing was confirmed by a reduction of uncleaved PARP, pro-caspase 3 and Mcl1 (**Figure 2D**). An effective decrease of CK1α protein levels upon silencing (around 40-50% with both the techniques used) was confirmed (**Figure 2E**). As expected, IPTG treatment did not affect the expression level of CK1α and of pro- and anti-apoptotic proteins in wt Jeko-1 cells, confirming the efficacy/specificity of the silencing strategy. To evaluate the effect of CK1α silencing on MCL cell proliferation, we performed cell cycle analysis of Jeko-1 wt and CK1α–silenced clones treated with IPTG (**Figure 2F**). The pro-apoptotic effect of CK1α knockdown was confirmed by the observation of a substantial increase in the fraction of CK1α-directed inducible shRNA-bearing Jeko-1 cells in the sub-G_1_ (apoptotic) phase of the cell cycle upon IPTG treatment. Alongside with the prevalent accumulation of sub-G1 (likely apoptotic) cells, CK1α silencing in the same cellular clone determined a minimal accumulation of cells in G_0_/G_1_ phase and a more evident reduction of cells in the S phase, suggesting an impairment also of proliferation upon CK1α knockdown. As expected, IPTG treatment of wt cells did not affect cell cycle. Moreover, CK1α silencing, through IPTG treatment for 6 days and for 13 days caused a reduction in the total count of live cells as judged by Trypan blue exclusion assay analysis, indicating a proliferation dysregulation (**Figure 2G**).

### CK1α Sustains Chronic Active BCR Dependent Signaling Pathways

**Figure 3:**
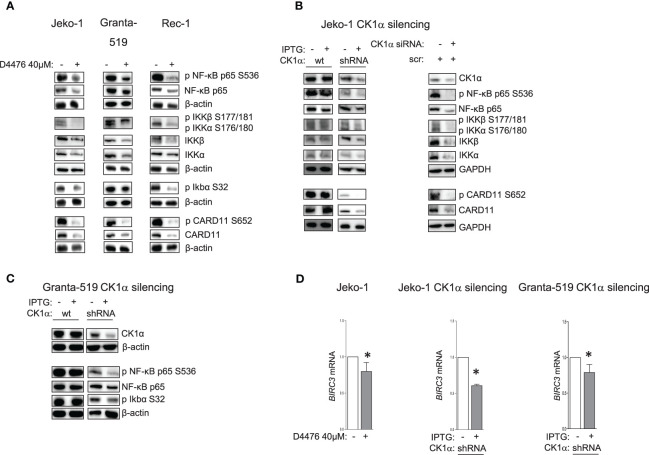
CK1α sustains NF-kB signaling pathway. Representative WB of NF-ĸB dependent signaling in MCL cell lines Jeko-1, Granta-519 and Rec-1, treated with D4476 40uM for 48h **(A)**, in Jeko-1 IPTG inducible *CSNK1A1* directed shRNA clone (named CK1α shRNA) and in Jeko-1 wt treated with IPTG 500µM for 13 days (**B** left panel) or Jeko -1 cells electroporated with *CSNK1A1* directed siRNAs (**B** right panel), or in Granta-519 IPTG inducible *CSNK1A1* directed shRNA clone (named CK1α shRNA) treated with IPTG 500µM for 72h **(C)**. Membranes for WB analysis were probed with antibodies listed in the figure. GAPDH or β actin were used as loading control. Experiments were repeated at least three times **(D)**. *BIRC3* mRNA expression in Jeko-1 treated with D4476 40uM for 48h, (left panel) or in Jeko-1 IPTG inducible *CSNK1A1* directed shRNA clone treated with IPTG 500µM for 13 days (middle panel) or in Granta-519 IPTG inducible *CSNK1A1* directed shRNA clone treated with IPTG 500µM for 7 days (right panel). Data represent mean ± SD of at least 3 independent experiments. * indicates *p* < 0.05.

CK1α is known to be involved in the regulation of several survival signaling pathways associated with BCR to which MCL cells are addicted for their growth. Bidere et al. (21) defined CK1α as a “conditionally essential malignancy” gene demonstrating that CK1α is required for constitutive NF-κB pathway in DLBCL. To understand if this holds true also for MCL cells, we treated three different MCL cell lines with D4476 40μM or DMSO for 48h and analyzed the NF-κB signaling pathway, in particular the activating phosphorylation of CARD11 on Ser 652, of NF-κB p65 on Ser 536, of the upstream IKKα/β, and of IkBα on Ser 32. Remarkably, the results showed a significant reduction of the activation of the NF-κB pathway upon CK1 chemical inhibition (**Figure 3A**). Most importantly, the specific effects of the inhibition on NF-κB were confirmed also by RNAi, both with IPTG-inducible shRNA transduced Jeko-1 and Granta-519 cells, and with electroporation of ds *CSNK1A1*-directed siRNA in Jeko-1 cells (**Figures 3B, C**). Surprisingly, CK1α inactivation not only caused a decrease in the activating phosphorylations of CARD11 on Ser 652, NF-κB-p65 on Ser 536, IKKα/β on Ser 176/180, Ser 177/181, but also in their total protein levels. Consequently, CK1α loss-of-function determined a reduced mRNA expression of the NF-κB-p65 target *BIRC3* (**Figure 3D**). *BIRC3* encodes for c-IAP-2 (cellular inhibitor of apoptosis), which confers resistance to apoptosis.

**Figure 4:**
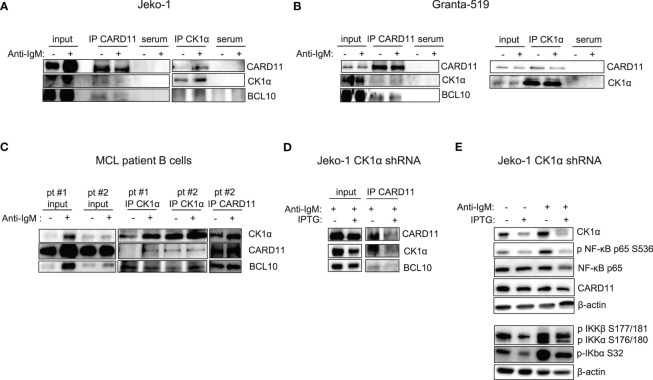
CK1α associates with the CBM1 complex, and its silencing reduces basal and anti-IgM induced NF-kB activation. **(A)** Immunoprecipitation (IP) of CARD11 and CK1α in Jeko-1 **(A)**, Granta- 519 **(B)**, purified B lymphocytes from two MCL patients (pt#1 and pt#2) **(C)**, stimulated with anti-IgM 10µg/ml for 5 min (serum= preimmune serum). **(D)** CK1α silencing reduces the binding of CK1α to CARD11 and BCL10 in the CBM1 complex in anti-IgM stimulated Jeko-CK1α shRNA inducible clone. Untreated and IPTG treated (500µM for 72h) Jeko-1 cells from the CK1α shRNA cellular clone were stimulated with anti-IgM 10µg/ml for 5 min and IP was carried out using CARD11 antibody. **(E)** CK1α silencing affects basal and anti-IgM stimulated NF-κB activation. Cells were treated as in **(D)**. WB was probed with antibodies listed in the figure. β-actin was used as loading control.

It has previously been demonstrated that CK1α controls canonical NF-κB activation in stimulated T and in DLBCL cells, participating to the activation of the upstream CBM1 complex (CARD11, BCL10, MALT1), through the phosphorylation of CARD11 and MALT1 (21, 22). We therefore evaluated a possible interaction between CK1α and the CBM1 complex in MCL cells. We stimulated Jeko-1, Granta-519 and MCL patients B cells with anti-IgM to fully activate the BCR and NF-κB signaling, and we performed immunoprecipitation and immunofluorescence experiments using CARD11 and CK1α antibodies. We found that CK1α associates with the CBM1 complex at basal and anti-IgM stimulated conditions (**Figures 4A–C**), as evidenced by coimmunoprecipitation of CK1α and CARD11 and as suggested by cellular colocalization studies of CK1α with CARD11 and MALT1 (**Figure S2**). Of note, CK1α silencing in Jeko-1 determined a reduction of CARD11 and BCL10 protein levels (**Figure 4D**) accompanied by a decrease in basal and anti-IgM-induced NF-κB pathway activation (**Figure 4E**). Of note, the perturbation of NF-κB signaling is achieved also within 72h (shorter) incubation time with IPTG treatment (silencing), indicating an acute/direct effect of CK1α silencing.

**Figure 5:**
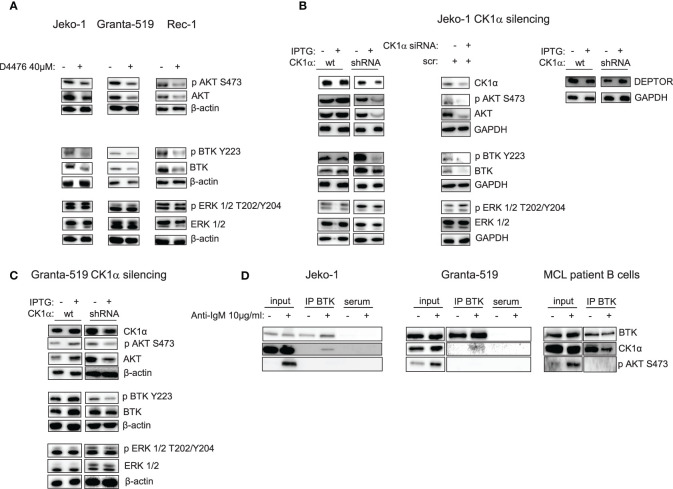
CK1α inactivation reduces AKT and BTK signaling pathways activation without affecting ERK/MAPK kinase and CK1α interacts with BTK in MCL cells. Representative WB of AKT, BTK and ERK1/2 dependent signaling in MCL cell lines Jeko-1 Granta-519 and Rec-1 treated with D4476 40uM for 48h **(A)** or in Jeko-1 IPTG inducible *CSNK1A1* directed shRNA clone (named CK1α shRNA) and in Jeko-1 wt treated with IPTG 500µM for 13 days (**B** left and right panel) or Jeko -1 cells electroporated with *CSNK1A1* directed siRNAs (**B** middle panel), or in Granta-519 IPTG inducible *CSNK1A1* directed shRNA clone (named CK1α shRNA) treated with IPTG 500µM for 72h **(C)**. Membranes for WB analysis were probed with antibodies listed in the figure. GAPDH or β actin was used as loading control. Experiments were repeated at least three times. **(D)** Immunoprecipitation (IP) of BTK in Jeko-1 (left panel), Granta-519 (middle panel), or purified B lymphocytes from one MCL patient (pt#2) (right panel), stimulated with anti-IgM 10µg/ml for 5 min. WB was probed with anti-CK1α and anti-BTK antibodies. To ensure BCR activation AKT phosphorylation on S473 (pAKT S473) was detected after anti-IgM stimulation in the total cell lysate (input).

As previously mentioned, PI3K/AKT is an essential cascade constitutively active in a subset of MCL, including all the aggressive blastoid variants and in MCL cell lines (28). It is known that CK1α may impinge on the PI3K/AKT pathway (17). Therefore, we sought to investigate whether CK1α could regulate this cascade also in MCL. CK1 chemical inhibition with D4476 in Jeko-1, Granta-519, Rec-1 MCL cells (**Figure 5A**) and CK1α silencing through the IPTG inducible method in Jeko-1 and Granta-519 led to a reduction of the phosphorylation of AKT on Ser 473 and of total AKT (**Figures 5B, C**). Wild-type Jeko-1 or Granta-519 cells treated with IPTG did not show any changes in the phosphorylated/total AKT protein content. It is known that CK1α phosphorylates the mTOR inhibitor DEPTOR, leading to its proteasomal degradation. As expected, as a consequence of CK1α silencing, DEPTOR level increased (**Figure 5B**). The reduction in AKT activating cascade is present also in *CSNK1A1* directed siRNA transiently transfected Jeko-1 cells (**Figure 5B**).

Since CK1α may have a broader regulative role on the BCR-transduced signal, we next investigated whether CK1α inhibition could affect other BCR-triggered events, such as BTK activation. **Figure 5** shows that both the BTK activating phosphorylation on Tyr 223 and the total amount of the BTK protein are downregulated when CK1α is chemically inhibited or silenced, indicating that CK1α could sustain BTK activity in MCL cells.

The observed CK1α modulation of some BCR dependent signaling cascades was present also at early time points of D4476 treatment, such as 6h (activating phosphorylation of AKT and CARD11) and 24h (activating phosphorylation of AKT and BTK) (**Figure S3**). To exclude a global rewiring of survival signaling events downstream the BCR as a direct effect of CK1α inhibition dependent cytotoxicity, and to selectively ascribe the effects observed on NF-κB, AKT and BTK cascades directly to the activity of CK1α, we also evaluated the effects of CK1α inactivation on ERK/MAP kinase signaling. To note, neither the chemical inhibition nor silencing of CK1α modified ERK 1,2 activating phosphorylation on Thr 202, Tyr 204, (**Figures 5** and **S3**), pointing to a role of CK1α in sustaining specifically NF-κB, AKT and BTK BCR dependent cascades. Altogether, the observation of early signaling changes upon CK1α inactivation indicates that they are very unlikely due to the ongoing apoptotic process. To investigate the mechanism of CK1α dependent regulation of BTK and NF-κB p65 stability, we checked whether the effects observed upon CK1α inactivation were due to transcriptional or post-translational mechanisms. Interestingly, we found that the mRNA expression of *BTK* and *REL A (p65)* did not change upon CK1α chemical inhibition or silencing, suggesting a post-translational mechanism of regulation of these two proteins (**Figures S4A, B**). We next asked whether this mechanism could be proteasome- or caspase-dependent. To this aim, the CK1α-directed shRNA transduced Jeko-1 clone was treated with Bortezomib (7.5nM), a clinically used proteasome inhibitor or with Z-VAD-FMK (2μM), a cell permeant pan-caspase inhibitor, along with IPTG to silence CK1α. Remarkably, p65 and BTK protein levels remained downregulated with the association of CK1α and bortezomib, while the CK1α silencing dependent reduction was rescued by Z-VAD-FMK treatment, suggesting that BTK and p65 degradation might be due to a caspase- rather than to a proteasome-dependent mechanism (**Figure S4C**).

Given the novel, unanticipated potential role of CK1α in regulating BTK function, we analyzed if these two kinases could physically interact. To this aim, we treated MCL cells with anti-IgM for 5 min and BTK was subsequently immunoprecipitated. Strikingly, CK1α was present in the immunocomplex (**Figure 5D**). To test specificity and BCR activation, phosphorylated AKT on Ser 473, indicative of fully activated BCR, was evaluated. Interestingly, CK1α physically interacts with BTK also at basal condition in patient isolated B cells from a particularly aggressive form of MCL. Thus, CK1α physically interacts with BTK downstream from the BCR in MCL cells.

### CK1α Inactivation Empowers BCR Inhibitor-Induced Cytotoxic Effects on MCL

**Figure 6:**
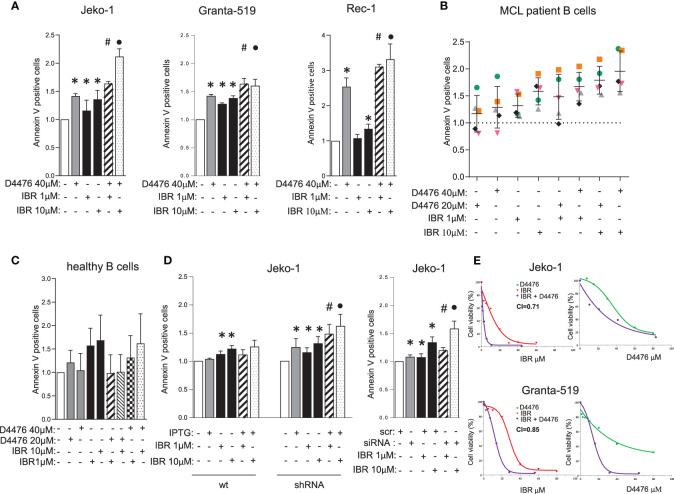
CK1α inactivation empowers Ibrutinib induced cytotoxicity. **(A–D)** Annexin V staining and FACS analysis of MCL and healthy B cells in which CK1α inactivation was associated with Ibrutinib (IBR) treatment. MCL cell lines Jeko-1, Granta-519, Rec-1 **(A)**, were treated with D4476 40 µM for 48h, B cells derived from MCL patients (n=5, **B**), or healthy B cells (n=4, **C**), were treated with D4476 20 µM or D4476 40 µM for 24h. From A to C, Ibrutinib 1 µM or 10 µM, alone or in combination with D4476 was added for the last 24h. Jeko-1 wt or Jeko-1 IPTG inducible *CSNK1A1* directed shRNA clone (named CK1α shRNA) were treated with IPTG 500µM for 13 days (**D** left panel), Jeko-1 wt cells were electroporated with *CSNK1A1* directed siRNAs oligo for 48h (**D** right panel), and treated with Ibrutinib 1 µM or 10 µM, alone or in combination with CK1α silencing for the last 24h. Data represent the mean ± SD of at least three independent experiments. * indicates p < 0.05; # indicates p < 0.05 between samples treated with Ibrutinib 1 µM together with D4476 (or CK1α silencing) and Ibrutinib 1 µM or D4476/CK1α silencing alone; • indicates *p* < 0.05 between samples treated with Ibrutinib 10 µM together with D4476 (or CK1α silencing) and Ibrutinib 10 µM or D4476/CK1α silencing alone. **(E)** Synergistic effect of D4476 and Ibrutinib in reducing cell viability. Dose response curve of Jeko-1 (upper) and Granta-519 (bottom) incubated for 72 hours with increasing concentrations of D4476 alone, (green squared curve), of Ibrutinib alone (red tringle curve), and with the combination of D4476 and Ibrutinib (purple squared curve). Cell viability was assessed with MTT test and reported as percentage over untreated cells. In Jeko-1, IC50 for D4476 alone was 41.5μM and for Ibrutinib alone was 11µM. IC50 for D4476 used in combination with Ibrutinib was 24μM, while IC50 for Ibrutinib used together with D4476 was 1.6μM. The CI between D4476 and Ibrutinib was calculated as to be 0.71. In Granta-519, IC50 for D4476 alone was 32μM and for Ibrutinib alone was 28µM. IC50 for D4476 used in combination with Ibrutinib was 14μM, while IC50 for Ibrutinib used together with D4476 was 12μM. The CI between D4476 and Ibrutinib was calculated as to be 0.85.

Given the aforementioned role of CK1α on BCR related signaling cascades, we next asked whether CK1α could interfere with BCR inhibitors (such as Ibrutinib or Duvelisib) -induced MCL cell apoptosis. Since we showed an unprecedented role of CK1α in sustaining BTK levels and activity and given the known role of CK1α on the BTK downstream CBM1 complex, we reasoned that its inhibition or silencing combined with direct BTK targeting would produce cooperative/synergic cytotoxic effects. Therefore, we treated three MCL cell lines, primary MCL B cells and healthy B lymphocytes with different doses of D4476 and Ibrutinib, alone or in combination. Annexin V staining and FACS analysis showed a cooperative effect of D4476 and Ibrutinib in inducing cell death in MCL cells (**Figures 6A, B** and **S5A**). This did not hold true for normal B cells, which were spared by the cytotoxic activity of D4476 (**Figure 6C**). The therapeutic potential of the association of Ibrutinib with CK1α inactivation was confirmed also in the models of CK1α silencing. Treatment with IPTG of *CSNK1A1*-directed shRNA transduced Jeko-1 clones significantly empowered Ibrutinib induced apoptosis (**Figures 6D** and **S5B**). Of note IPTG-treated wt Jeko-1 cells did not show increase in apoptosis compared to Ibrutinib-only treated cells, confirming the efficacy and the specificity of the silencing method. These results were confirmed also in experiments of nucleofection of *CSNK1A1-*directed siRNAs in Jeko-1 cells (**Figure 6D**). The rise in apoptosis in the combination treatments was confirmed by immunoblot analysis of PARP cleavage (**Figure S5C**). Moreover, MTT viability assays in the presence of increasing concentrations of Ibrutinib, D4476 or the combination of the two drugs for 72 h showed a strong synergic effect of the two compounds, as judged by the calculated Combination Index (CI) below 1 (**Figure 6E**). Of note, the synergy was present also in Granta-519 cells, a line known to be less sensitive to Ibrutinib (IC50 in Granta-519 28 µM *versus* 11 µM for Jeko-1), suggesting that CK1α inhibition could partly overcome Ibrutinib resistance.

**Figure 7:**
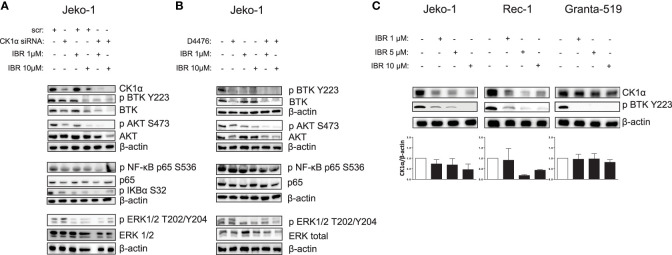
Ibrutinib cooperates with CK1 inactivation in modulating BCR dependent signaling cascades, reducing also CK1α expression. **(A, B)** Representative WB of NF-kB p65, BTK, AKT, ERK1/2 dependent phosphorylation and total protein expression in Jeko-1 cells electroporated with *CSNK1A1* directed siRNA for 48h **(A)**, and in Jeko- 1 exposed to D4476 40 µM for 48h **(B)**, treated with Ibrutinib (IBR) 1 µM or 10 µM for the last 24h. β actin was used as loading control. The figure shows a representative WB, that was performed on at least 3 independent experiments. **(C)** Representative WB (upper panel) and densitometric analysis (lower panel) of CK1α protein expression in Jeko-1, Rec-1, Granta-519 MCL cells, treated with Ibrutinib 1µM, 5µM and 10 µM for 24h, of three (Jeko-1), two (Rec-1), four (Granta-519) independent experiments. β actin was used as loading control.

Analysis of intracellular signaling revealed that Ibrutinib cooperated with CK1 inactivation (both chemical inhibition with D4476 and gene silencing) in modulating NF-ĸB, AKT and BTK-dependent cascades by further reducing the levels of phosphorylated NF-ĸB-p65 on Ser 536, AKT on Ser 473 and BTK on Tyr 223, without affecting ERK1/2 MAP kinase activity (**Figures 7A, B**). To note, we noticed that in Ibrutinib-treated Jeko-1 cells CK1α expression was decreased. We therefore treated all the MCL cell lines available with different concentrations of Ibrutinib for 24h. Strikingly, Ibrutinib effectively caused a reduction of CK1α protein expression in Jeko-1, Rec-1 and - even if to a lower extent - in Granta-519 cells (**Figure 7C**). The effectiveness of Ibrutinib was confirmed by the abolition of phosphorylated BTK on Tyr 223.

We next tested the interaction of CK1α inhibition with Duvelisib, a PI3Kγ/δ inhibitor approved for the treatment of Chronic Lymphocytic Leukemia (CLL) and Follicular Lymphoma (FL). However, its use in MCL is still under scrutiny (29, 30).

**Figure 8:**
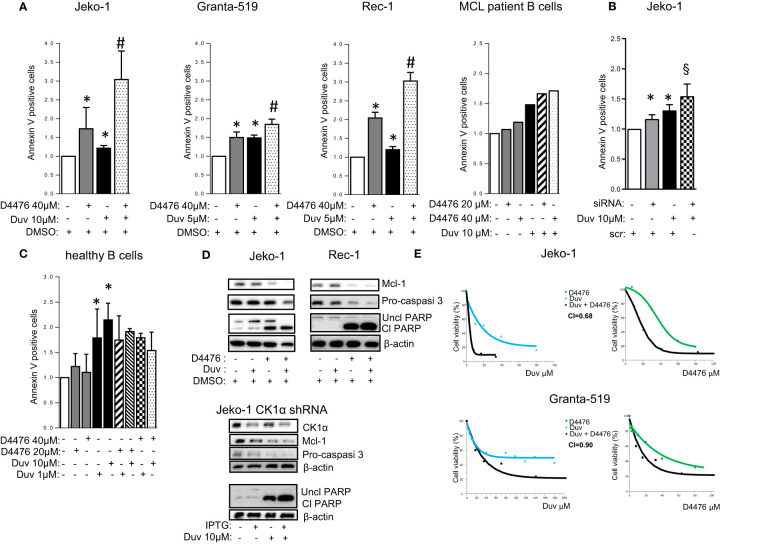
CK1α inactivation empowers Duvelisib induced cytotoxicity. **(A–C)** Annexin V staining and FACS analysis of MCL and healthy B cells in which CK1α inactivation was associated with Duvelisib (Duv) treatment. **(A)**, MCL cell lines Jeko-1, Granta-519, Rec-1, were treated with Duv for 48h and with D4476 for the last 24h at the indicated concentrations. Purified B cells derived from 1 MCL patient were treated with Duv and D4476 for 24h. **(B)** Jeko-1 wt cells were electroporated with *CSNK1A1* directed siRNAs for 48h and treated with Duv 10µM alone or in combination with CK1α silencing for the last 24h. **(C)** Healthy B cells (n=4) were treated with Duv 1µM or 10µM, D4476 20µM or 40µM or the combination of both compounds for 24h. Data represent the mean ± SD of at least n=6 (Jeko-1), n=9 (Granta-519), n=3 (Rec-1) independent experiments normalized over control. * indicates p < 0.05 compared to DMSO treated cells, ^#^ indicates p < 0.05 between samples treated with Duvelisib together with D4476 and Duvelisib or D4476 alone; § indicates p < 0.05 between samples treated with Duvelisib together with CK1α silencing and Duvelisib or CK1α silencing alone. **(D)** Representative WB analysis of Mcl1, Procaspase-3, and PARP cleavage in Jeko-1 and Rec-1 treated with Duv (10 µM for Jeko-1 and 5µM for Rec-1) for 48h and with D4476 40 µM for the last 24h (upper panel) and Jeko-1 IPTG inducible *CSNK1A1* directed shRNA clone (named CK1α shRNA) treated with IPTG 500µM for 13 days and with Duv 10 µM for the last 24h, (lower panel). CK1α antibody was used to monitor the silencing efficacy. β actin was used as loading control. The figure shows a representative WB, that was performed on at least 3 independent experiments. **(E)** Synergistic effect of D4476 and Duv in reducing cell viability. Dose response curve of Jeko-1 (upper) and Granta-519 (bottom) incubated for 72 hours with increasing concentrations of D4476 alone, (green squared curve), of Duv alone (light blue star curve), and with the combination of D4476 and Duv (black filled circle curve). Cell viability was assessed with MTT test and reported as percentage over untreated cells. In Jeko-1, IC50 for D4476 alone was 41.5μM and for Duv alone was 16,85µM. IC50 for D4476 used in combination with Duv was 18,63μM, while IC50 for Duv used together with D4476 was 3,95 μM. The CI between D4476 and Duv was calculated as to be 0.68. In Granta-519, IC50 for D4476 alone was 32μM and for Duv alone was 71µM. IC50 for D4476 used in combination with Duv was 14,41 μM, while IC50 for Duv used together with D4476 was 32,27μM. The CI between D4476 and Ibrutinib was calculated as to be 0.90.

We first verified the efficacy of Duvelisib on a panel of MCL cell lines and purified B cells from six MCL patients. Cells were treated with different doses of Duvelisib for 24 and 48 h. Duvelisib caused MCL cell apoptosis in a dose and time dependent manner. The efficacy of the drug was monitored by assessing the levels of phosphorylated AKT on Ser 473, which were always reduced in all the cells treated (**Figure S6**). Next, the effects on apoptosis of the combination of CK1α inhibition together with Duvelisib was analyzed through Annexin V staining and FACS analysis. Jeko-1, Granta- 519, Rec-1 MCL cells were treated with D4467 and Duvelisib alone or in combination (**Figures 8A** and **S7**). Also, Jeko-1 CK1α silenced cells were treated with Duvelisib (**Figure 8B**). The combination of Duvelisib and CK1α inactivation cooperatively increased the cytotoxicity induced by the single agents. Of note, this cooperation was absent in healthy normal B cells, which were spared by D4476-induced cytotoxicity (**Figure 8C**). The higher rate of apoptosis obtained in the combination experiments was confirmed also by immunoblot analysis of PARP, pro-caspase 3 and Mcl1 protein cleavage (**Figure 8D**). The effect was synergic since the calculated combination index was lower than 1 (**Figure 8E**). Of note, CK1α inactivation caused a significant reduction of the IC50 of Duvelisib in the more resistant Granta-519 cells (**Figure 8E**). Interestingly, as observed with Ibrutinib, treatment with Duvelisib at different concentrations caused a reduction of CK1α protein expression in MCL cells (**Figure S8**).

## Discussion

In this work we have provided evidence that the Ser/Thr kinase CK1α is a pivotal regulator of the BCR cascades and may be targeted to enhance the cytotoxic effects of BCR inhibitors, suggesting the rationale for innovative therapeutic strategies for patients with MCL in association with novel agents.

Our findings suggest that CK1α has a pro-survival role in MCL cells, through the regulation of BCR-linked signaling cascades and protects from Ibrutinib/Duvelisib-induced apoptosis indicating that CK1α could be a novel targetable molecule in this malignancy. We described here that CK1α inactivation disrupts the activity of specific critical cascades, namely NF-κB, PI3K/AKT, BTK, which sustain BCR addiction in MCL. Therefore, CK1α inhibition may be a powerful way to boost BCR inhibitors -mediated cell death in MCL.

We showed here that the expression of CK1α, even with a variable outcome, was elevated in most of MCL patients’ B cells and in all the MCL cell lines analyzed compared to B cells from healthy controls. At variance with normal B cells, we found an atypical nuclear diffuse microspeckled localization in MCL cells, similar to what we already observed in MM (17). Several reports have established that CK1α is associated with distinct structures and factors in the cell and has a cell-cycle-dependent subcellular distribution (31). The nuclear diffusion of CK1α in MCL and MM samples suggests that this localization could be a feature of CK1α in B-cell derived tumors. CK1α might have nuclear roles dependent on the transformed status. However, at present there are no data that allow to speculate whether this kinase takes part in the regulation of gene transcription, DNA metabolism or chromatin dynamics in MCL.

We also demonstrated that CK1α sustains MCL survival and proliferation. The alterations of the cell cycle and the arrest of proliferation, seen upon CK1α silencing, suggest that CK1α has a role in cell division. Indeed it has been previously demonstrated that CK1α might participate in mitosis regulating spindle placing (32). Moreover, phosphoproteomics data suggest a putative regulative role for CK1α in phosphorylation of about 50% of mitosis-related phosphopeptides (33).

In addition, the prosurvival effects of CK1α might depend on its known role in the regulation of the intensity of NF-κB activation downstream the BCR (21, 22) and, as suggested by our observation in MM cells, in the upholding of the PI3K/AKT pathway (17). The relevance of these two pathways in MCL are well-established. Aberrant NF-κB activation is associated with maintenance and progression of a range of lymphoid malignancies, including MCL (34, 35). Moreover, a constitutive activation of the PI3K/AKT pathway contributes to the pathogenesis of MCL likely due to loss of PTEN expression (28). Ibrutinib resistance in CLL and DLBCL could be attributed to aberrant activation of the AKT pathway (36). Indeed, initial reports indicate that targeting PI3K may overcome Ibrutinib intolerance/resistance in CLL (37) and Richter syndrome (38).

Indeed, our experiments demonstrated that CK1α sustains the NF-kB dependent signaling (**Figure 3**). Inhibition or silencing of CK1α led to a reduction of both phosphorylated and total NF-kB p65. The level at which CK1α seems to act in the NF-kB signaling is upstream of p65, since phosphorylation and total IKKα/IKKβ protein levels were also reduced. Importantly, the demonstration that CK1α inhibition/silencing significantly causes a reduction of CARD11 phosphorylation and levels and that CK1α is present in the CBM1 complex, both at resting and at BCR-stimulated conditions (**Figure 4**), suggests that this kinase might regulate the IKK/NF-κB activation by sustaining the activity of CARD11, as shown in DLBCL (22).

Moreover, we have also provided evidence that CK1α regulates other pivotal signaling molecules in MCL cells (**Figure 5**). Indeed, CK1α inhibition or silencing led to a reduction of AKT phosphorylation on Ser 473 and total AKT and of BTK phosphorylation on Tyr 223 and total BTK. Instead, the MAPK/ERK pathway was unaffected. The regulative role of CK1α on the PI3K/AKT signaling axis was already documented in previous works. For instance, CK1α is known to phosphorylate DEPTOR, targeting it for proteasomal degradation (39). This would leave mTOR free to phosphorylate AKT, with the consequent activation of the downstream survival pathway. The inhibition of the kinase would therefore cause an increase in DEPTOR activity. However, the reduction of AKT expression could be also a consequence of p53 activation upon CK1α inhibition, since previous work established that cells undergoing p53-dependent apoptosis downregulate AKT in a caspase-dependent mechanism (40). Along this line, we showed that in MM CK1α inactivation caused a p53/caspase dependent reduction of AKT (17). Thus, the observed AKT decrease upon CK1α inactivation in MCL cells could still rely on the inhibitory effects exerted by the unleashed p53/caspase axis.

Remarkably, we have also demonstrated an unanticipated novel role of CK1α on BTK function. CK1α chemical inactivation and gene silencing caused a reduction of activating phosphorylation and of total BTK levels. It has been previously reported that BTK is needed for BCR-induced activation of NF-κB (41, 42) and BTK dependent phosphorylation of IKBα is associated to NF-κB-p65 nuclear translocation and activation, independently from IKK (43). On the other hand, the activity of the NF-κB subunit p65, which directly interacts with the BTK promoter, may cause an increase of BTK activity in B cells (44). Our findings suggest that CK1α could be involved in the autoregulatory loop between BTK and NF-κB in MCL. Mechanistically, we provide evidence that CK1α might control BTK protein stability through a post-translational mechanism that does not involve the proteasome but instead relies on caspases (**Figure S4**). Remarkably, we also showed that CK1α physically interacts with BTK, thus making it possible that CK1α facilitates BTK activating phosphorylation on Tyr 223. Even if it needs to be further investigated, this new finding is particularly relevant in the context of the therapy of MCL and other BTK-addicted NHL. To this regard, our data indicate that the molecular effects of CK1α on BTK and PI3K/AKT might impinge on the action of different classes of BCR inhibitors. Indeed, CK1α loss of function synergically potentiated Ibrutinib or Duvelisib cytotoxicity, compared to single treatments, in all the models tested, including cells less sensitive to Ibrutinib and Duvelisib (Granta-519 cells). Our results showed that CK1α inhibition could boost the Ibrutinib dependent NF-κB and AKT inactivation, impinging on crucial survival pathways important for MCL clonal expansion such as AKT, NF-κB and BTK. This is particularly meaningful since it has been shown that Ibrutinib resistance is associated to an increase of mTOR and NF-κB mediated signaling (9). CK1α-dependent activation of these pathways could be therefore usefully targeted in a therapeutic perspective and the design of novel inhibitors of CK1α would be suitable for a use in the clinical setting to overcome MCL therapy resistance. Indeed, a successful example of such an approach is the development of the dual PI3Kδ/CK1ϵ inhibitor Umbralisib, which has shown clinical efficacy in relapsed/refractory CLL and lymphomas, alone, or in combination with BTK inhibitors (45–47).

We have herein provided compelling evidence that CK1α sustains chronic active BCR-linked signaling cascades in MCL, namely the AKT, NF-κB and BTK-dependent pathways, and promotes tumor survival and proliferation. CK1α genetic and chemically inhibition causes MCL cell death and synergically empowered Ibrutinib and Duvelisib induced apoptosis. Our results are particularly meaningful in the perspective of developing novel strategies that abrogate BCR activation. Therefore, CK1α may be a key target opening new perspectives in the treatment of MCL patients, especially those with relapsed/refractory disease.

## Data Availability Statement

The original contributions presented in the study are included in the article/**Supplementary Material**. Further inquiries can be directed to the corresponding authors.

## Ethics Statement

The studies involving human participants were reviewed and approved by Ethic Committee of the Padova University Hospital internal Institutional Board. The patients/participants provided their written informed consent to participate in this study.

## Author Contributions

SM and FP conceived, designed the experiments, and wrote the paper. SM, AF, LQT, ZS, and MC performed the experiments. SM, ZS, AF, MC, and FP analyzed the data. GSc, AV, GB, MP, AD, FV, RZ, CG, GSe, and LT provided patient samples and clinical data. SM, GSe, LT, and FP contributed reagents/materials/analysis tools. All authors contributed to the article and approved the submitted version.

## Funding

Supported by Italian Gilead Oncohematology Fellowship Program to FP, LT, FV, Associazione Italiana per la Ricerca sul Cancro (AIRC) to FP (IG 18387) and LT (IG 25024), PRIN (Progetti di rilevante interesse nazionale)-MIUR Prot. 2017ZXT5WR to SM, Ricerca per Credere nella vita (R.C.V) ODV to LT.

## Conflict of Interest

FP is in the Advisory Board of Roche and Janssen. LT reports grants, personal fees from Janssen, personal fees from Abbvie, grants from Gilead. AV is in the Scientific Board of Janssen and Takeda and in the Speaker Bureaux of Janssen, Italfarmaco, Gilead and Abbvie. GS has reported consultancy or advisory board for Janssen and Celgene and has received research support from Roche and Novartis, all outside of the submitted work.

The remaining authors declare that the research was conducted in the absence of any commercial or financial relationships that could be construed as a potential conflict of interest.

## Publisher’s Note

All claims expressed in this article are solely those of the authors and do not necessarily represent those of their affiliated organizations, or those of the publisher, the editors and the reviewers. Any product that may be evaluated in this article, or claim that may be made by its manufacturer, is not guaranteed or endorsed by the publisher.
