# Supplementary material for: Protein Kinase CK1α Sustains B-Cell Receptor Signaling in Mantle Cell Lymphoma
Source: Front Oncol. 2021 Oct 14;11:733848. doi: 10.3389/fonc.2021.733848 (PMC8551451; doi:10.3389/fonc.2021.733848)
Supplement: Supplementary file 1 [file DataSheet_1.pdf]

Supplementary Material

Supplementary Figures

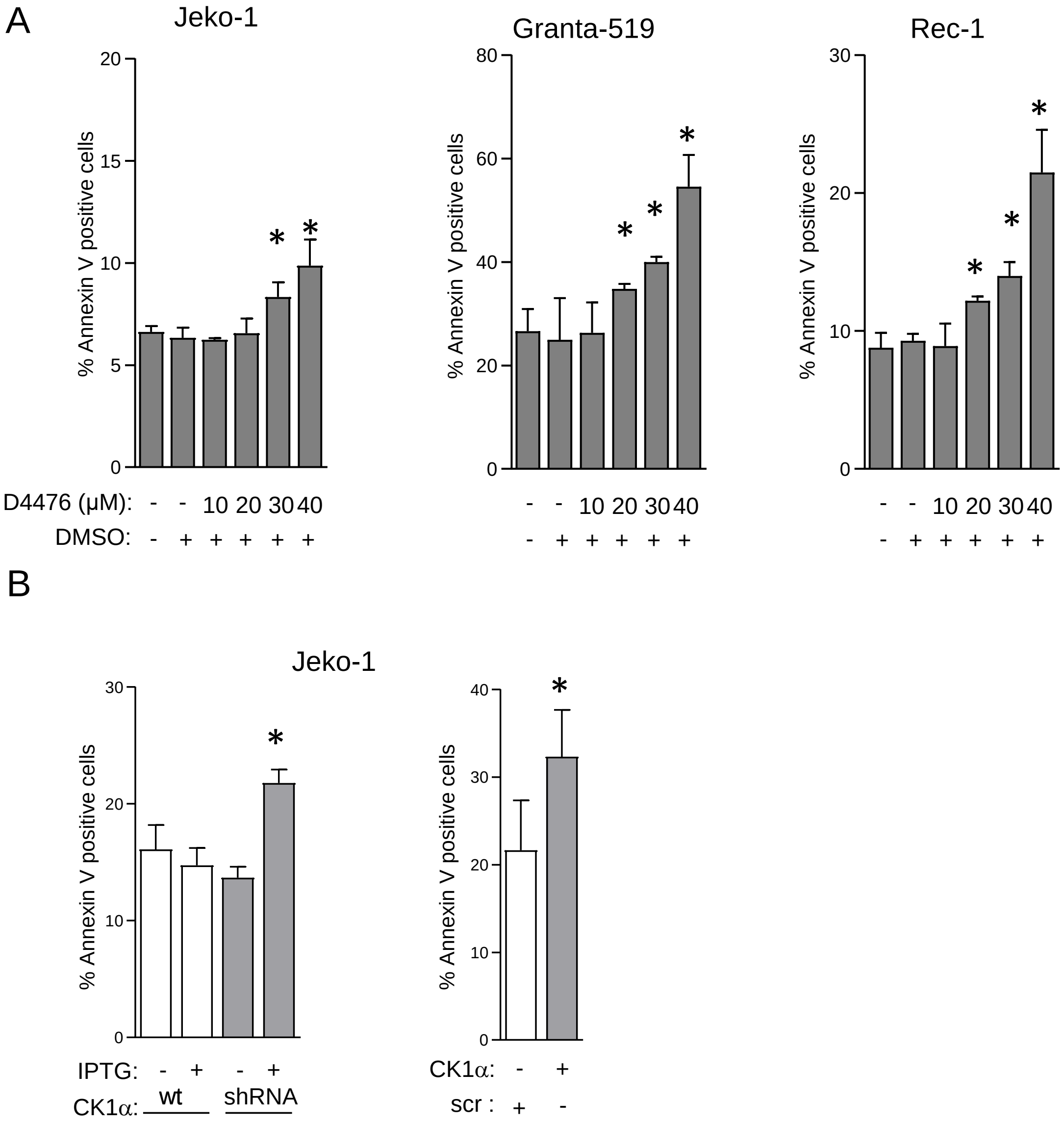

Supplementary Figure 1. CK1α sustains MCL cell survival. Histogram showing % of annexin V positive cells of MCL cell lines Jeko-1 Granta-519 and Rec-1 treated with different concentrations of D4476 for 48h (A), Jeko-1 IPTG inducible *CSNK1A1* directed shRNA clone (named CK1α shRNA) and Jeko-1 wt treated with IPTG 500µM for 13 days (B left panel) or Jeko-1 cells electroporated with *CSNK1A1* directed siRNAs (B right panel). Data are expressed as mean ±SD of n=3 (A), n=4 (B left panel) and n=3 (B right panel) independent experiments. * indicates $p < 0.05$ compared to the untreated cell population.

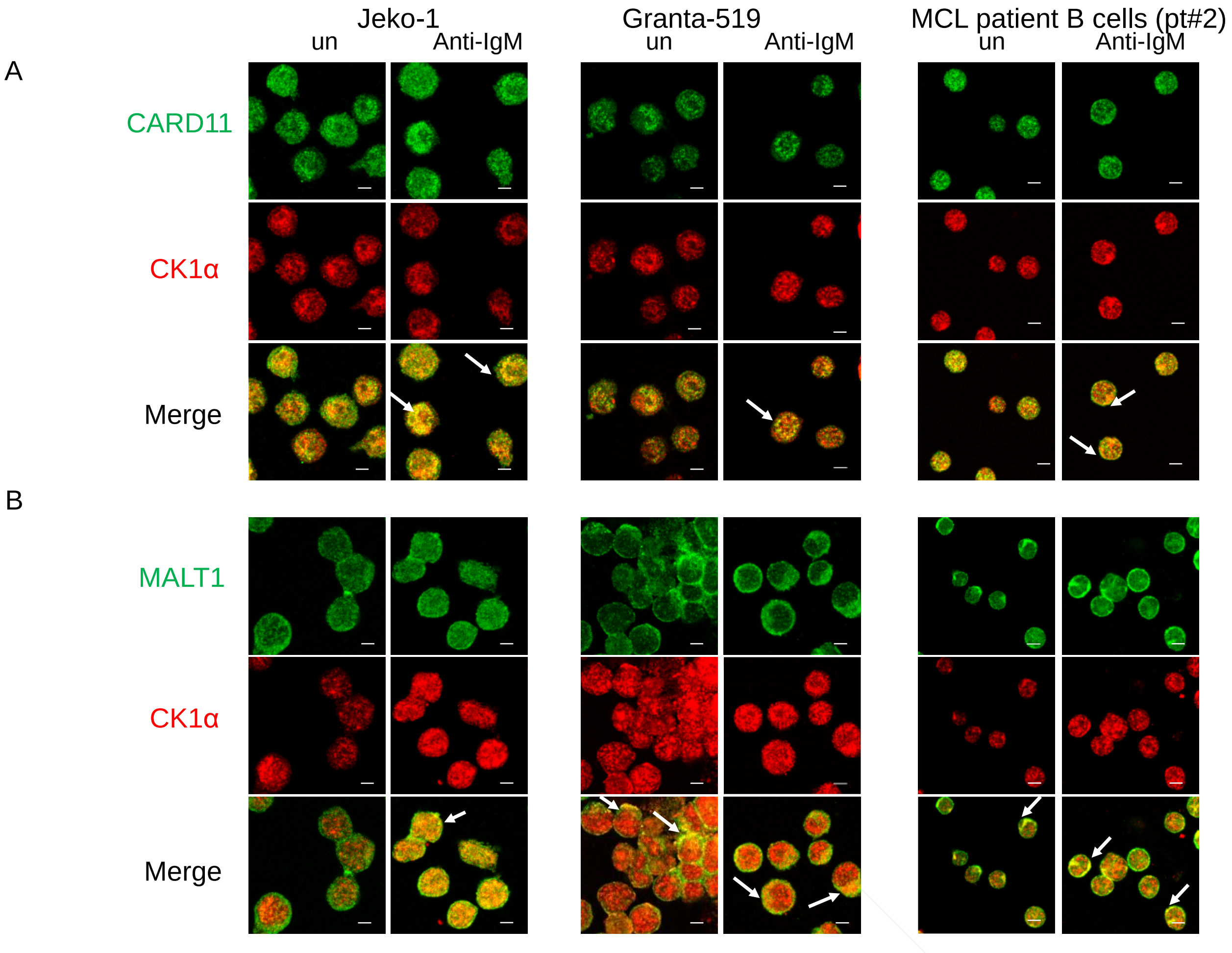

Supplementary Figure 2. CK1α associates with the CBM complex. Co-localization through immunofluorescence of CK1α (red) with CARD11 (green) (A) and MALT1 (green) (B) in Jeko-1 (left panel), Granta-519 (middle panel) and purified B lymphocytes from one MCL patient (pt#2, right panel), stimulated with anti IgM 10µg/ml for 5 min. Images have been acquired with 63x oil objective. Scale bar =5µm. Arrows indicate discrete zones of colocalization.

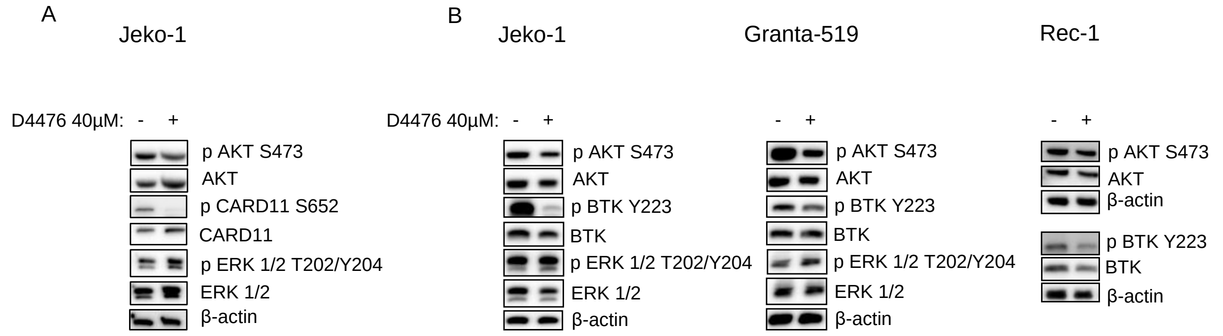

Supplementary Figure 3. Effects of short term CK1 inhibition on BCR signaling pathways. Representative WB of BCR dependent signaling in MCL cell lines Jeko-1 treated with D4476 40 µM for 6 h (A) and in Jeko-1, Granta-519 and Rec-1 treated with D4476 40 µM for 24 h (B). Membranes for WB analysis were probed with antibodies listed in the figure. β actin was used as loading control.

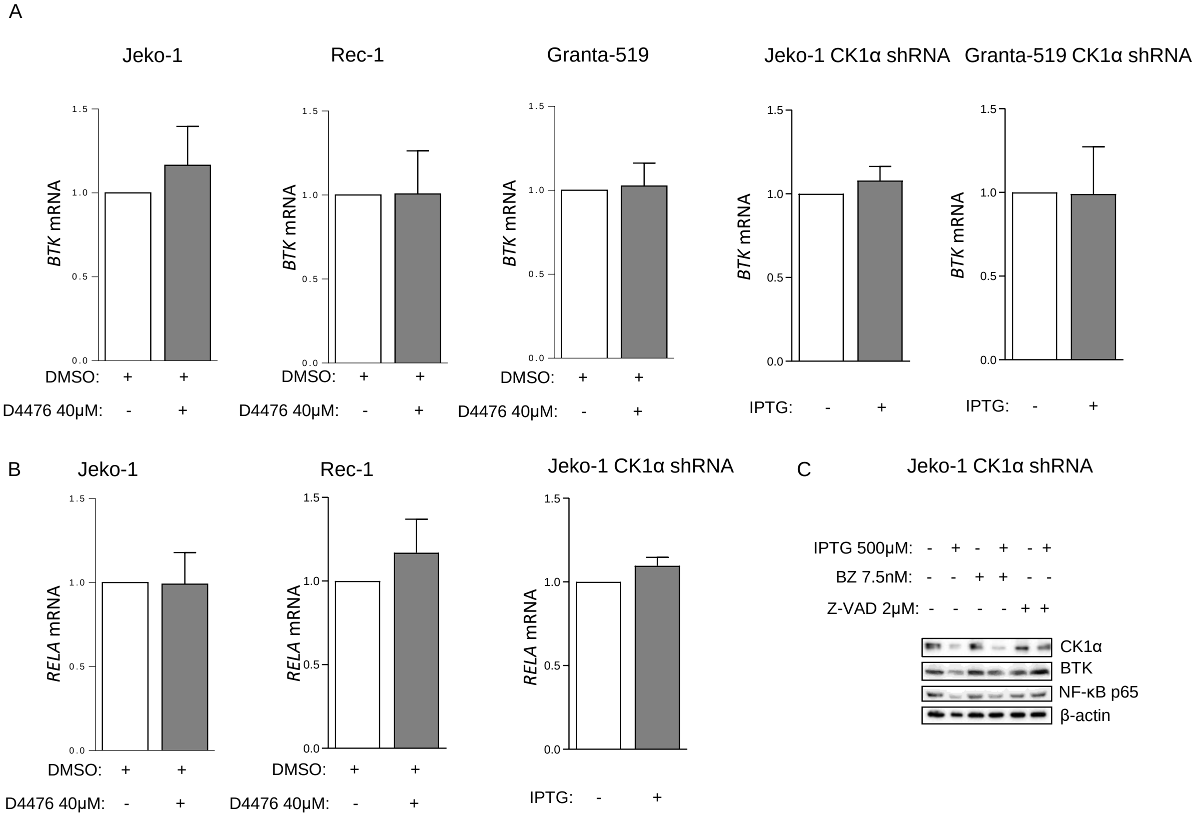

Supplementary Figure 4. CK1α inactivation reduces BTK and NF-kB total protein level through a post-translational mechanism. (A-B) CK1α chemical inhibition or silencing does not change *BTK* or *RELA* mRNA expression**.** Quantitative Real Time PCR analysis of *BTK* (A) and *RELA* (B) mRNA expression performed in Jeko-1, Rec-1, Granta-519 treated with D4476 for 48h, on Jeko-1 cells from the CK1α shRNA cellular clone (Jeko-1 CK1α shRNA) treated with IPTG 500µM for 13 days, and on cells from Granta-519 CK1α shRNA cellular clone (Granta-519 CK1α shRNA) treated with IPTG 500µM for 7 days. Data represent mean ± SD of at least 3 independent experiments. (C) Representative

WB of BTK and NF-kB p65 in Jeko-1 IPTG inducible CK1α directed shRNA clone treated with IPTG 500µM for 13 days (to silence CK1α) alone, or in association with bortezomib (BZ) and Z-VAD-FMK (Z-VAD). BZ 7.5 nM and Z-VAD 2µM were added for the last 24h before harvesting. β actin was used as loading control.

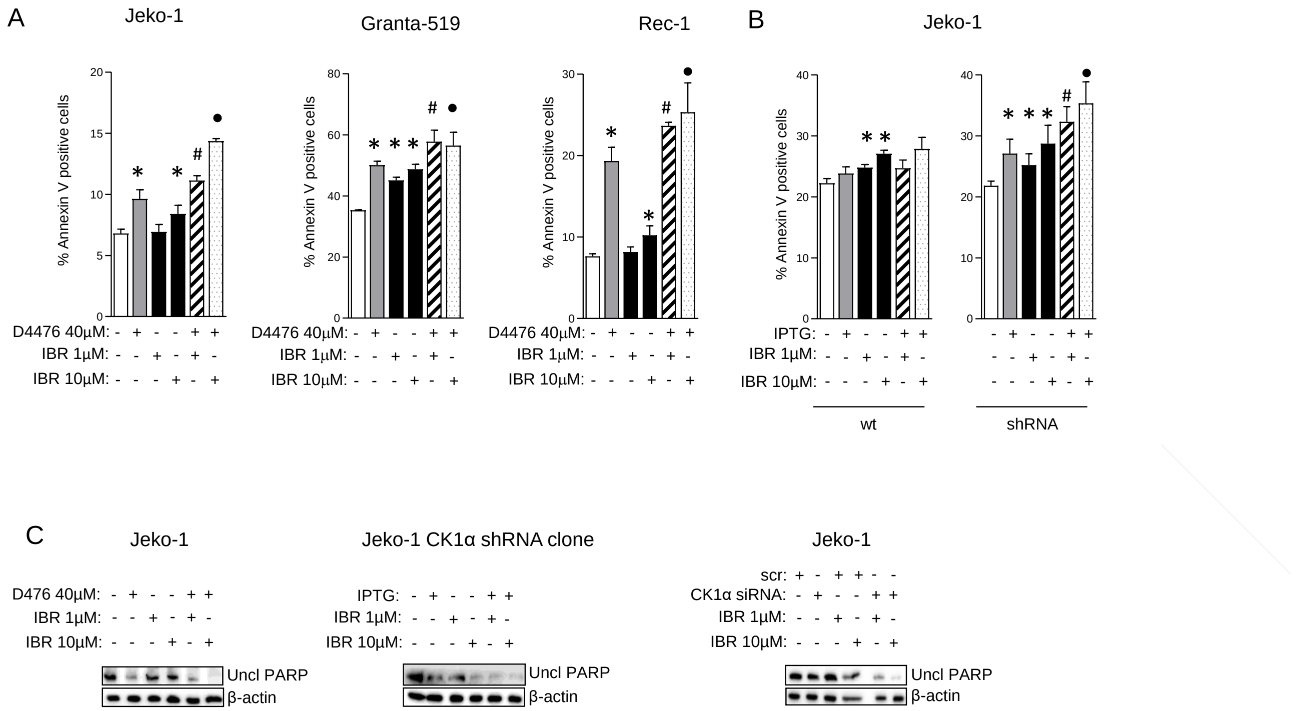

Supplementary Figure 5. CK1α inactivation empowers Ibrutinib induced cell death. (A) % of Annexin V positive MCL cell lines in which CK1α chemical inactivation was associated with Ibrutinib (IBR) treatment**.** Jeko-1, Granta-519, Rec-1 were treated with D4476 40 µM for 48h, Ibrutinib 1 µM or 10 µM, alone or in combination with D4476 was added for the last 24h. (B) % of Annexin V positive cells Jeko-1 wt or Jeko-1 IPTG inducible *CSNK1A1* directed shRNA clone (named CK1α shRNA) which were treated with IPTG 500µM for 13 days and with Ibrutinib 1 µM or 10 µM, alone or in combination with CK1α silencing for the last 24h. Data represent the mean of % annexin V positive cells ± SD of at least three independent experiments. * indicates $p < 0.05$; # indicates $p < 0.05$ between samples treated with Ibrutinib 1 µM together with D4476 (or CK1α silencing) and Ibrutinib 1 µM or D4476/CK1α silencing alone; • indicates $p < 0.05$ between samples treated with Ibrutinib 10 µM together with D4476 (or CK1α silencing) and Ibrutinib 10 µM or D4476/ CK1α silencing alone. (C) Representative WB of uncleaved PARP expression in CK1α silenced or CK1 chemical inhibited Jeko-1 MCL cells treated with Ibrutinib 1 µM or 10 µM for the last 24h. Left panel: Jeko-1 cells were treated as in A. Middle panel: the Jeko-1 CK1α shRNA clone was treated with IPTG 500µM for 13 days to silence CK1α. Right panel: Jeko-1 cells were electroporated with *CSNK1A1* directed siRNA oligo for 48h. In all panels ibrutinib 1 µM or 10 µM was added for the last 24h. β actin was used as loading control. The figure shows a representative WB, that was performed on at least 3 independent experiments.

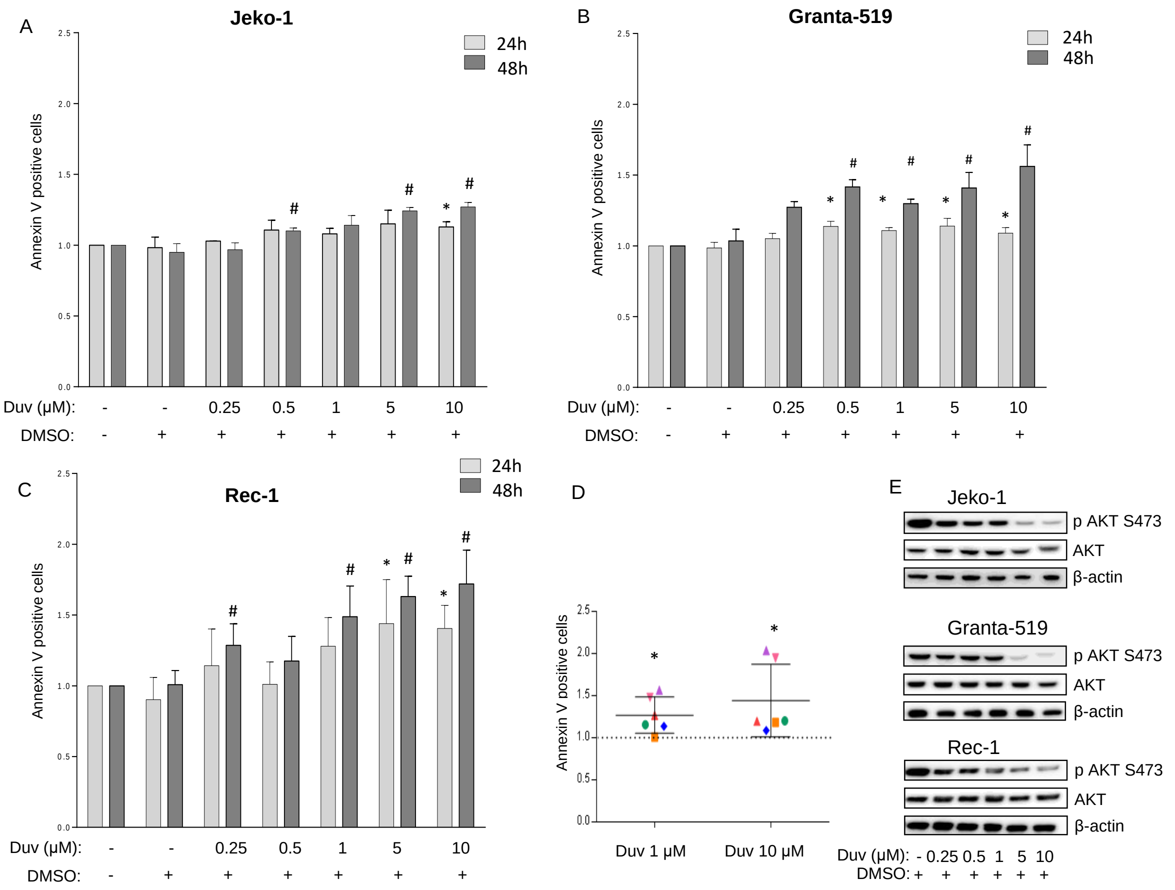

Supplementary Figure 6. Duvelisib induces MCL cell apoptosis. (A-D) Annexin V staining of MCL cell lines Jeko-1 (A), Granta-519 (B), Rec-1 (C), MCL patient derived B cells (D) treated with different concentration of Duvelisib (0.25 µM, 0.5 µM, 1µM, 5µM and 10 µM) for 24h (A, B, C, D) and 48h (A, B, C). Data are expressed as ratio over untreated and as mean ±SD of n=3 (A, B, C) independent experiments, and ratio over DMSO treated cells (n=6 patient samples) in D. * indicates $p < 0.05$ at 24h ; # indicates $p < 0.05$ at 48h. (E) Representative WB performed on three independent experiments of Ser 473 phosphorylated AKT (p S473 AKT), total AKT in Jeko-1 (upper panel), Granta 519 (middle panel) and Rec-1 (bottom panel) treated with increasing concentrations of Duvelisib for 48h. β actin was used as loading control.

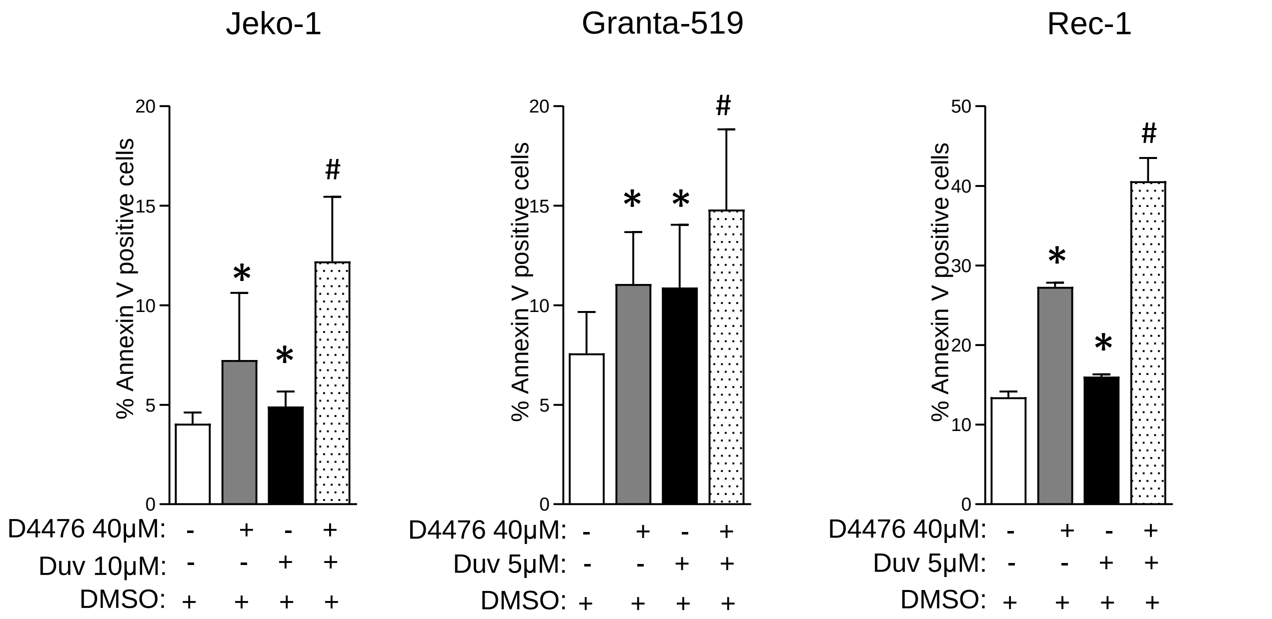

Supplementary Figure 7. CK1α inactivation empowers Duvelisib induced cell death. % of Annexin V positive MCL cell lines in which CK1α chemical inhibition was associated with Duvelisib (Duv) treatment**.** Jeko-1 cells were treated with Duv 10 µM and Granta-519, Rec-1 with Duv 5 µM for 48h. D4476 40 µM alone or in combination with Duv was added for the last 24h. Data represent the mean of % annexin V positive cells ± SD of at least three independent experiments. * indicates $p < 0.05$; # indicates $p < 0.05$ between samples treated with Duv together with D4476 and Duv or D4476 alone.

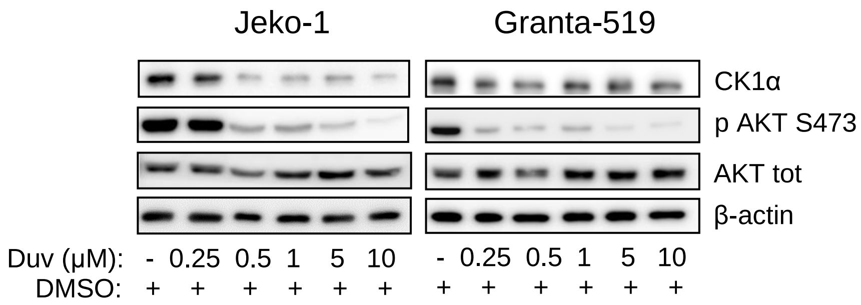

Supplementary Figure 8. Duvelisib reduced CK1α expression. Representative WB of CK1α protein expression in Jeko-1, Rec-1, Granta-519 MCL cells, treated with increasing concentration of Duv (0.25-10 µM) for 24h of three (Jeko-1), two (Granta-519) independent experiments. Phosphorylated AKT in S473 (p AKT S473) and total AKT was detected to confirm Duv efficacy. β actin was used as loading control
